# Differentiation of breast tuberculosis and breast cancer using diffusion-weighted, T2-weighted and dynamic contrast-enhanced magnetic resonance imaging

**DOI:** 10.4102/sajr.v22i2.1377

**Published:** 2018-10-25

**Authors:** Dibuseng P. Ramaema, Richard J. Hift

**Affiliations:** 1 Division of Radiation Medicine, Nelson R Mandela School of Medicine, University of KwaZulu-Natal, South Africa; 2 Division of Medicine, Nelson R Mandela School of Medicine, University of KwaZulu-Natal, South Africa

## Abstract

**Background:**

The use of multi-parametric magnetic resonance imaging (MRI) in the evaluation of breast tuberculosis (BTB).

**Objectives:**

To evaluate the value of diffusion-weighted imaging (DWI), T2-weighted (T2W) and dynamic contrast-enhanced magnetic resonance imaging (DCE-MRI) in differentiating breast cancer (BCA) from BTB.

**Method:**

We retrospectively studied images of 17 patients with BCA who had undergone pre-operative MRI and 6 patients with pathologically proven BTB who underwent DCE-MRI during January 2014 to January 2015.

**Results:**

All patients were female, with the age range of BTB patients being 23–43 years and the BCA patients being 31–74 years. Breast cancer patients had a statistically significant lower mean apparent diffusion coefficient (ADC) value (1072.10 ± 365.14), compared to the BTB group (1690.77 ± 624.05, *p* = 0.006). The mean T2-weighted signal intensity (T2SI) was lower for the BCA group (521.56 ± 233.73) than the BTB group (787.74 ± 196.04, *p* = 0.020). An ADC mean cut-off value of 1558.79 yielded 66% sensitivity and 94% specificity, whilst the T2SI cut-off value of 790.20 yielded 83% sensitivity and 83% specificity for differentiating between BTB and BCA. The homogeneous internal enhancement for focal mass was seen in BCA patients only.

**Conclusion:**

Multi-parametric MRI incorporating the DWI, T2W and DCE-MRI may be a useful tool to differentiate BCA from BTB.

## Introduction

Although breast cancer (BCA) is the most prevalent malignancy amongst South African women, accounting for 20.62%,^1^ the prevalence of breast tuberculosis (BTB) is by contrast much lower, with reported ranges between 0.3% and 0.4%.^2,3^ Breast tuberculosis is a manifestation of extra-pulmonary tuberculosis (EPTB), a condition which has become considerably more commonly encountered as a consequence of the human immunodeficiency virus (HIV) pandemic;^4,5^ yet, studies and guidelines on the management of EPTB frequently do not mention the breast as a potential site for tuberculosis.^6,7,8^ Given the infrequency with which BTB is diagnosed, little research has been performed on the disease. Breast tuberculosis and BCA may present clinically in a very similar fashion leading to potential misdiagnosis^9,10^; given that an accurate diagnosis is essential if the patient is to receive correct and timely treatment, this may be problematic.^8^

Conventional imaging with mammography and ultrasound has limited ability to differentiate between BTB and BCA.^11^ It is therefore appropriate to investigate the use of newer and more complex imaging modalities in the differentiation of BCA and BTB. Studies that have used magnetic resonance imaging (MRI) to differentiate EPTB from malignant lesions are those which focused on other body parts such as the central nervous system (CNS)^12,13,14^ and the musculoskeletal system.^15,16^ Peng et al. used the diffusion-weighted imaging (DWI) and magnetic resonance spectroscopy (MRS) to differentiate intracranial tuberculomas from high-grade gliomas and found significant differences. The diagnostic accuracy was higher when using the minimum apparent diffusion coefficient (ADC) value from the DWI than the maximum MRS ratios of Cho/Cr, Cho/NAA and Cho/Cho.^13^ Magnetic resonance imaging has also been utilised in pulmonary tuberculosis (PTB) to differentiate between tuberculous and malignant nodules.^17,18,19,20^ Only one study was found in the literature which aimed to differentiate BTB from BCA with the use of MRS in four BTB patients with no comparison group.^21^ The authors suggested that the absence of choline peak and the presence of a strong lipid peak favoured BTB rather than BCA.^21^

There are multiple studies that utilise DWI and ADC to distinguish malignant from benign breast disease.^22,23,24,25,26,27^ Diffusion-weighted imaging measures the microscopic movement of water molecules in biological tissues, with the pathologic processes altering their mobility; the detection of these changes aids in lesion characterisation.^28^ The ADC is derived from DWI sequences. Apparent diffusion coefficient values, measured in mm^2^/s, are calculated within a given area. This reflects water restriction. Tissues with high ADC values, because of increased diffusion, display a brighter signal, whereas those with lower ADC values appear darker, because of restricted diffusion.^29^ Most malignant lesions display lower ADC values when compared to benign or inflammatory lesions.^26^ Rong-Feng Qu et al. recently conducted a meta-analysis of the differential diagnosis of benign and malignant breast tumours. The authors found that the ADC values of normal breast tissues were higher than those of benign tissues, and that the values of benign lesions were higher than those of malignant tumours.^23^

Breast dynamic contrast-enhanced magnetic resonance imaging (DCE-MRI) involves administration of a magnetic compatible intravenous (IV) contrast agent in order to detect and characterise lesions.^30^ Further advantage is the ability to evaluate the breast lesion enhancement parameters and kinetic curves. It has been demonstrated that during the wash-in rate, malignant lesions demonstrated higher maximum enhancement when compared to benign lesions.^31^ Dynamic contrast-enhanced MRI kinetic characteristics have been shown to correlate with tumour neovascularity in which more vascular tumours demonstrate strong enhancement.^32^

The T2-weighted (T2W) MRI uses the tissue transverse relaxation times to generate the signal.^33^ The displayed T2 signal intensity (T2SI) is used in the characterisation of tissues and lesions.^33^ Studies that used the T2SI in the discrimination of benign and malignant breast lesions mainly analysed the morphologic T2SI appearance rather than the quantitative values.^34,35^ The majority of BCAs appear hypointense on T2W, compared to most benign lesions that appear hyperintense.^36^

The purpose of our study was to evaluate the use of the DWI parameter of ADC, the DCE-MRI enhancement morphologic characteristics and the T2W parameter of T2SI value to differentiate BCA from BTB.

## Patients and methods

We retrospectively identified 24 patients with histologically proven BCA (including ductal carcinoma in situ [DCIS]) who underwent DWI, T2W and DCE-MRI during January 2014 to December 2014. Seven patients did not have a full set of images on the picture archiving and communication system (PACS) and were excluded from the study, which resulted in the final total of 17 patients.

For the BTB group, we included six prospectively and consecutively identified patients with proven BTB who also underwent the DWI, T2W and DCE-MRI. Although five patients had conclusive histology from either the breast or the axillary lymph nodes, one patient had inconclusive histology from breast and axilla, but had proven concurrent PTB, for which she was receiving anti-tuberculous therapy (ATT). Her clinical and radiological features were compatible with BTB, and both the breast and the axillary nodes responded satisfactorily to ATT. All BTB patients had undergone screening investigations at the referring breast clinic, including ultrasonography, with or without mammograms, depending on their age. Following the histological confirmation of BTB, the patients were invited for a baseline MRI scan within 2 weeks of confirmation of the diagnosis. Scans were not performed within 2 weeks of a biopsy in order to avoid the presence of haematoma and/or inflammation confounding our results.

### Magnetic resonance imaging image acquisition

Magnetic resonance imaging images were retrospectively analysed following retrieval from the hospital’s PACS. Magnetic resonance imaging was performed on a 1.5 Telsa machine (Siemens, Erlangen, Germany) using a dedicated breast coil. The patients were scanned in the prone position. A power injection of 20 mL intravenous (IV) Magnevist^®^ (Gadopentetate Dimeglumine, Bayer) Standard 469 mg/mL (0.5 mmol/mL) at a dosage of 0.1 mmol/kg was administered to all the patients. The IV contrast rate was 3 mL/s followed by a 20 mL saline flush administered as a bolus. The T1 dynamic phase scan time was 6 min 41 s, during which five dynamic sequences were obtained in the axial position at various time points. The DWI images with the ADC map were also acquired during the same scan. The technical parameters are reported in Table 1.

**TABLE 1:** Local dynamic contrast-enhanced breast magnetic resonance imaging protocol technical parameters.

MRI sequence	Acquisition plane	TR (ms)	TE (ms)	Inversion time (ms)	Matrix size	FoV (mm)	Slice thickness (mm)	Voxel size (mm)
Localiser	sagittal	7.6	3.53	-	384 × 512	400	6	2.1 × 1.6 × 6.0
T1 pre-contrast GE 3D	axial	8.6	4.70	-	299 × 384	320	1	1.0 × 0.7 × 1.0
T1 GE 3D dynamic sequences (1 pre-contrast and 5 post-contrast)	axial	9.1	4.76	-	299 × 284	340	1.5	1.1 × 0.9 × 1.5
T1 3D Dixon	axial	7.20	First 2.38; second 4.76	-	320 × 320	340	1.8	1.1 × 1.1 × 1.8
T1 fat saturated	axial	680	10	-	224 × 320	320	4	1.4 × 1.0 × 4.0
T2 STIR	axial	5600	59.0	170	314 × 320	340	4	1.1 × 1.1 × 4.0
T2 TSE	axial	6100	111	-	384 × 512	320	4	1.7 × 1.3 × 4.0
DWI between values 0 and 800 s/mm^2^	axial	9200	86	180	150 × 192	380	4	2.0 × 2.0 × 4.0

MRI, magnetic resonance imaging; TR, repetition time; TE, echo time; FoV, field of view; DWI, diffusion-weighted imaging; TSE, turbo spin echo; GE, gradient echo; STIR, short tau inversion recovery.

### Magnetic resonance imaging image analysis

All the images were read by one of the authors who is a radiologist with more than 10 years’ experience in breast radiology. The following qualitative parameters were recorded:

- The BCA pathological subtype from the pathology records.
- The DCE-MRI parameters were classified using the Breast Imaging Reporting and Data System (BI-RADS) descriptors^37^ and included the following:
  ■ *The enhancement pattern*: Either mass-like enhancement (MASS) or non-mass enhancement (NME).
  ■ *Non-mass enhancement distribution pattern*: Focal, linear, segmental, regional, multiple regions or diffuse.
  ■ *Non-mass enhancement internal enhancement*: Homogeneous, heterogeneous, clumped and clustered ring.
  ■ *MASS internal enhancement*: Homogeneous, heterogeneous, rim enhancement and dark internal septations.
- The presence or absence of axillary nodes was added to the above descriptors.

We further recorded the ADC values (×10^−6^ mm^2^/s) derived from the DWI images, and T2SI value derived from the T2W. For the quantitative parameters, the region of interest (ROI) was drawn on three contiguous axial images around the lesion circumference and the average recorded. All images were analysed on the dedicated Syngo-via (Siemens, Erlangen, Germany) reading platform.

### Statistical analysis

Data were entered into an Excel spreadsheet (*Microsoft Excel 2013*. Redmond, WA) and analysed using the STATA software package (StataCorp. 2015: Stata Statistical Software, Release 14. College Station, TX). Differences in ADC value and T2SI between the two groups were assessed with a two-tailed unpaired *t*-test. Differences in ADC value and T2SI with enhancement pattern matching were assessed using analysis of variance (ANOVA). The results were considered significant where *p* < 0.05.

## Ethical consideration

Ethical clearance was obtained from the institution’s Biomedical Research Ethics Committee (BREC) (Reference number BF213/13).

## Results

### Patient characteristics

All the patients were female. The ages of the BTB patients ranged from 23 to 43 years, and for those with BCA, 31–74 years. Three BTB patients were HIV positive, one HIV negative and two not tested. The BCA subtypes included 13 cases of invasive ductal cancer (IDC) (76.5%); 3 cases of DCIS (17.6%), of whom one had high-grade DCIS; and 1 case (5.9%) of invasive lobular cancer (ILC).

### Radiologic findings

**FIGURE 1:**
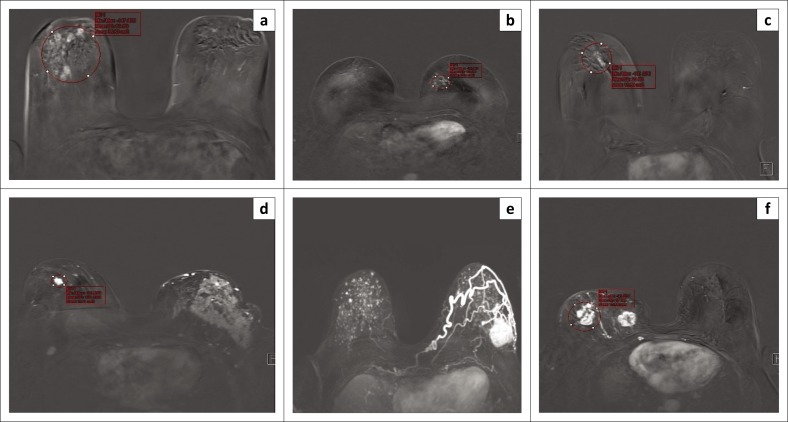
Dynamic contrast-enhanced magnetic resonance imaging enhancement characteristic of various patients with breast cancer: (a) clustered ring, (b) clumped, (c) linear distribution, (d) homogeneous (e) larger homogeneous pattern and (f) multicentric heterogeneous enhancement.

**FIGURE 2:**
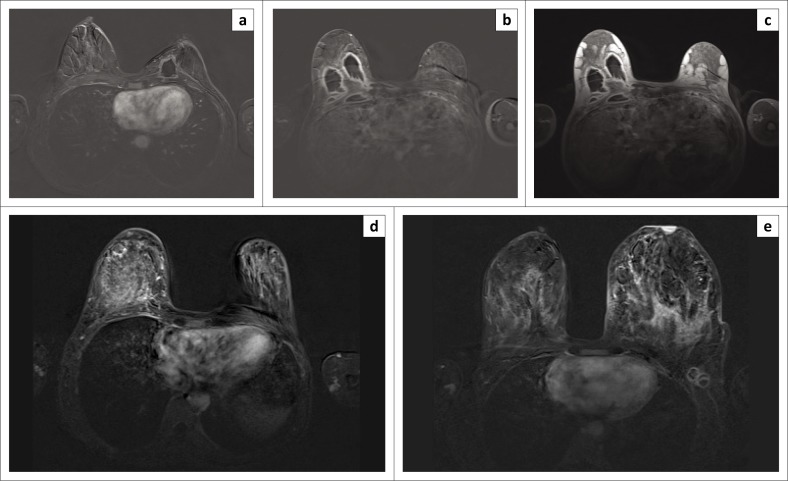
Dynamic contrast-enhanced magnetic resonance imaging enhancement patterns of various patients with breast tuberculosis: (a) focal rim, (b) multiple regions rim, (c) same patient as (b) to better demonstrate multiple rim enhancement on the non-subtracted T1-weighted image, (d) clumped diffuse and (e) clumped diffuse associated with rim-enhancing left axillary nodes.

The MRI morphological enhancement characteristics of the BCA patients are shown in Figure 1 and those of BTB in Figure 2.

#### Mass versus non-mass enhancement pattern

In the patients with BTB, 3/6 (50%) had mass-like enhancement, and 3/6 (50%) had NME patterns.

In the patients with BCA, 11/17 (65%) had mass-like enhancement, and 6/17 (35%) had NME patterns.

#### Enhancement patterns

Amongst the BTB patients, 3 (50%) had clumped diffuse NME, 2 (33%) showed focal mass rim and 1 (17%) demonstrated multiple regions mass rim. Axillary nodes in BTB patients demonstrated rim enhancement (Figure 2).

Of the BCA patients, 5 (29%) had heterogeneous enhancement, 7 (41%) had homogeneous enhancement, 4 (24%) had clumped enhancement and 1 (6%) had clustered ring enhancement (Figure 1).

#### Apparent diffusion coefficient and T2-weighted signal intensity values

**FIGURE 3:**
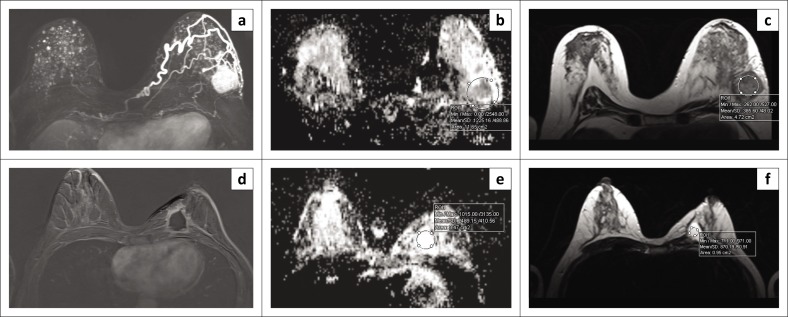
Apparent diffusion coefficient value and the T2 signal intensity measurements. (a–c) Axial dynamic contrast-enhanced magnetic resonance imaging (DCE-MRI), diffusion-weighted imaging (DWI) and T2-weighted (T2W)-MRI images of a 61-year-old female patient with left breast cancer: (a) Axial T1 post-contrast subtracted image demonstrates strongly homogeneous enhancing mass with neovascularity in the left breast. (b) Corresponding axial DWI-MRI apparent diffusion coefficient (ADC) map image shows dark signal. (c) Axial T2-weighted image shows the tumour hypointensity. (d–f) Axial DCE-MRI, DWI and T2W-MRI images of a 41-year-old female patient with left breast tuberculosis: (d) Axial T1 post-contrast subtracted image demonstrates a hypointense rim-enhancing mass in left breast; (e) Corresponding axial DWI-MRI ADC map image demonstrates a bright signal; and (f) Axial T2-weighted image shows a uniformly hyperintense signal.

The representative images demonstrating data extraction for ADC values and T2SI measurements of the BCA and BTB patients are depicted in Figure 3.

We found significant differences in both ADC and mean T2SI values between the two groups. The mean ADC for BTB was 1690.8 ± 624.1, and for BCA, it was 1072.1 ± 365.1 (*p* = 0.006). The mean T2SI for BTB was 787.7 ± 196.0, and for BCA, it was 521.6 ± 233.7 (*p* = 0.020) (Table 2).

**TABLE 2:** Comparison of the diffusion-weighted imaging and T2-weighted magnetic resonance imaging quantitative parameters for the breast tuberculosis and breast cancer patients.

Parameter	BTB (*n* = 6)		BCA (*n* = 17)		*p*
	Mean ± SD	95% CI	Mean ± SD	95% CI	
ADC (×10^−6^ mm^2^/s)	1690.8 ± 624.1	1035.9–2345.7	1066.3 ± 375.5	873.2 – 1259.4	0.008
T2SI value	787.7 ± 196.0	582.0–993.5	523.5 ± 240.8	399.7-647.3	0.025

ADC, apparent diffusion coefficient; BCA, breast cancer; BTB, breast tuberculosis; T2SI, T2 signal intensity value.

**FIGURE 4:**
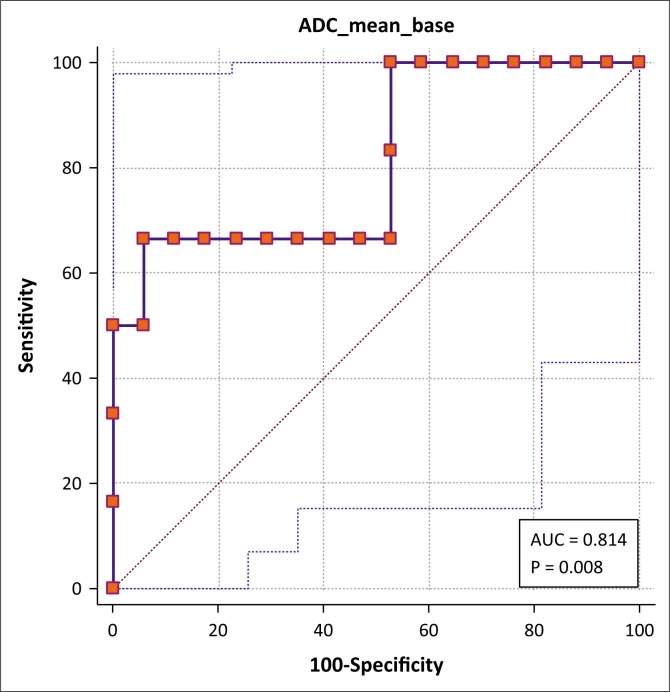
Preliminary receiver operating characteristic curve analysis of the apparent diffusion coefficient value of the breast lesions for differentiating cancer from tuberculosis.

With regard to the ADC values, receiver operating characteristic (ROC) curve analysis showed a sensitivity of 66.7% (CI 22.3% – 95.7%) and specificity of 94.1% (CI 71.3% – 99.9%) for a diagnosis of BTB at a cut-off value of 152.2 × 10^6^ mm^2^/s, with an area under the curve (AUC) of 0.81 (CI 0.60% – 0.94%) (Figure 4).

**FIGURE 5:**
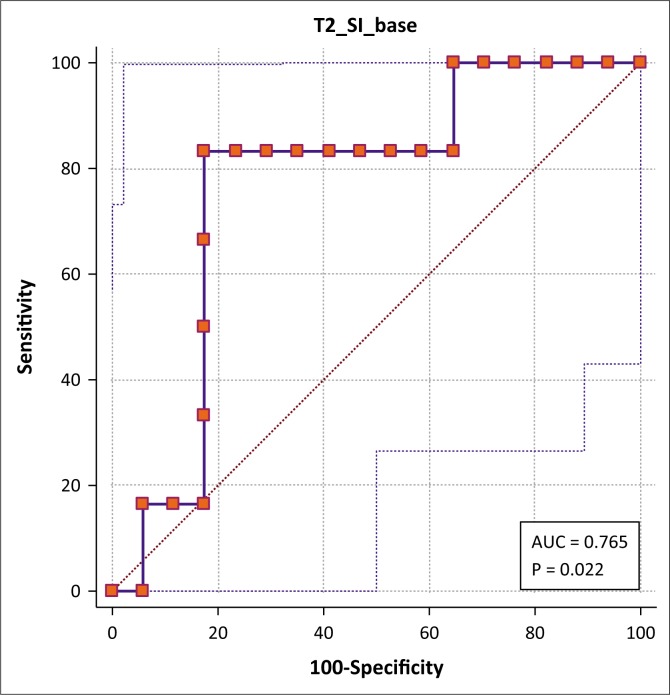
Preliminary receiver operating characteristic curve analysis of the T2 signal intensity of the breast lesions for differentiating cancer from tuberculosis.

The corresponding values for T2SI were 83.3% specificity (CI 35.9% – 99.6%) and 82.4% sensitivity (CI 56.6% – 96.2%) at a cut-off value of 670.6, with an AUC of 0.77 (CI 0.54% – 0.91%) (Figure 5).

#### Breast cancer versus breast tuberculosis with matched patterns

These differences persisted when the two groups were matched for the patterns of mass-like enhancement (MASS) and non-mass enhancement (NME) and compared with ANOVA. Significance was shown for both mean ADC value (*p* = 0.040) and T2SI (*p* = 0.015).

## Discussion

This study evaluated the ability of the two quantitative MRI markers, the ADC value and the T2SI, and the qualitative dynamic post-contrast enhancement patterns to discriminate between BCA and BTB.

### Utility

We have shown that BCA patients have significantly lower mean ADC and T2SI values compared to BTB patients. ROC curve analysis suggests that the ADC value is a better discriminator between BCA and BTB; however, the confidence intervals are unacceptably wide and further validation, using a larger sample, is required before these can be accepted as having real diagnostic value.

### Explanation

This outcome is explicable in that malignant lesions have high cellularity with resulting restricted diffusion, yielding lower ADC values, a parameter derived from the DWI. This finding has been shown to be consistent across many studies,^23,24,25,26,27,38,39^ with few exceptions.^12^ Woodhams et al.^24^ found malignant breast tumours to have lower ADC values than benign lesions; furthermore, the IDC displayed lower values when compared to the non-invasive ductal cancer (NIDC). In contrast, Chatterjee et al.^12^ found similar ADC values for brain tuberculomas and brain metastases.

The studies that utilised T2SI to discriminate the benign from the malignant breast lesions predominantly focused on the qualitative T2 signal morphologic appearance rather than the quantitative value as in our study.^34,35^ The data on the use of quantitative T2SI as a discriminator between benign and malignant breast lesions are scanty.^40^ Other studies in which T2SI quantitative values were used in differentiating benign from malignant lesions involve other parts of the body and not the breast.^41,42^ Henz et al.^41^ investigated pulmonary nodules with MRI using the quantitative parameters ADC and T2SI in a granulomatous endemic area. They found both the mean T2SI ratio and the ADC to be significant in differentiating the benign from the malignant pulmonary nodules.

Post-contrast morphological analysis revealed that the homogeneous internal enhancement pattern was exclusively observed in BCA patients. This qualitative feature can be used as a further descriptor for separating the two conditions when other imaging or pathology results are equivocal. The diffuse distribution of BTB was visually associated with enlargement of the breast; although this appearance can be confused with inflammatory BCA, the presence of rim-enhancing axillary nodes is more supportive of BTB than BCA.^43^

## Conclusion

Based on our findings, we conclude that the combination of DCE-MRI morphologic enhancement pattern, the T2SI and the quantitative DWI ADC values may provide some useful non-invasive information in distinguishing malignant from non-malignant illness (in this case, BTB) in patients with suspicious breast lesions. Development of accurate diagnostic algorithms will require accumulation of a larger patient database for BTB in particular; more broadly, however, continued investigation is appropriate in terms of the development of tests to distinguish malignant from benign disease more generally. As with mammographic and ultrasonic findings, the MRI findings alone are not sufficient to distinguish BTB and BCA with acceptable accuracy, and histology remains essential for this purpose.
